# Association of insulin resistance with near peak bone mass in the femur and lumbar spine of Korean adults aged 25-35: The Korean National Health and Nutrition Examination Survey 2008-2010

**DOI:** 10.1371/journal.pone.0177311

**Published:** 2017-07-13

**Authors:** Min Soo Choo, Se Rin Choi, Jun Hyun Han, Seong Ho Lee, Young Suk Shim

**Affiliations:** 1 Department of Urology, Hallym University Medical Center, Hallym University College of Medicine, Chuncheon, Seoul, Korea; 2 Department of Pediatrics, Hallym University Medical Center, Hallym University College of Medicine, Chuncheon, Seoul, Korea; University of Hull, UNITED KINGDOM

## Abstract

**Objective:**

This study aimed to evaluate the relationship between insulin resistance and the bone mineral density (BMD) of femur and lumbar spine in Korean adults who are expected to exhibit near peak bone mass.

**Methods:**

Data from the Korean National Health and Nutrition Examination Survey 2008–2010 were analyzed. A total of 2,750 participants aged 25−35 years were included. Insulin resistance was assessed using a homeostatic model assessment of insulin resistance (HOMA-IR) and serum fasting insulin.

**Results:**

In a multivariate linear regression analysis, the HOMA-IR was significantly inversely associated with the BMD of the total hip (TH, β = −0.052, *P* = 0.002), femoral neck (FN, β = −0.072, *P*<0.001), femoral trochanter (FTr, β = −0.055, *P* = 0.003), femoral intertrochanter (FITr, β = −0.041, *P* = 0.015), and lumbar spine (LS, β = −0.063, *P* = 0.001) among all study subjects after adjustment for gender, age, height, weight, whole body fat mass percentage, systolic blood pressure, diastolic blood pressure, total cholesterol, triglyceride, high-density lipoprotein cholesterol, low-density lipoprotein cholesterol, vitamin D, smoking, alcohol intake, physical activity, education level, and household income in both genders as well as labor, the use of oral contraceptives, and age at menarche in females. The serum fasting insulin was significantly inversely associated with the BMD of the TH (β = −0.055, *P* = 0.001), FN (β = −0.072, *P*<0.001), FTr (β = −0.055, *P* = 0.003), FITr (β = −0.045, *P* = 0.009), and LS (β = −0.064, *P* = 0.001) among all subjects in a multivariate linear regression analysis.

**Conclusion:**

Our results suggest that insulin resistance may be independently and inversely associated with the near peak bone mass of the femur and lumbar spine.

## Introduction

From a metabolic perspective, bone is an active connective tissue that is continuously deposited and resorbed throughout its lifetime. Specifically, approximately 40% of bone mass is developed during adolescence, and 90% of bone mass is deposited by age 18; subsequently, peak bone mass is attained by the time the individual reaches their late twenties [1]. This bone mineral accretion in childhood and adolescence is related to long-term bone health, and bone mass accrued throughout childhood is an important determinant of life-long skeletal health and osteoporosis risk. Higher peak bone mass is associated with a greater protective factor against fracture and osteoporosis [2].

Insulin resistance is defined as a decreased tissue response to insulin-mediated cellular actions, and this condition is the inverse of insulin sensitivity [3]. Insulin resistance plays a central role in the metabolic disturbances associated with obesity, metabolic syndrome (MetS), and type 2 diabetes mellitus (T2DM) [4]. Increased insulin levels compensate for the reduced responsiveness of target cells. Studies have reported that insulin resistance with compensating hyperinsulinemia may be related to increased bone mass [5,6,7] because insulin exerts an anabolic effect on bone formation [8]. Individuals with T2DM, which is considered to be linked to increased insulin resistance, exhibit higher bone mineral density (BMD) [9]. However, the relationship between insulin resistance and BMD should be considered after adjusting for BMI or fat mass because T2DM is closely related to obesity or an increased body mass index (BMI). Recently, some studies have suggested that the positive association between insulin resistance and BMD are not observed after adjusting for confounding factors. Hyperinsulinemia has been demonstrated to positively correlate with BMD, but this relationship was not significant after adjusting for body mass index (BMI) [10]. These results suggest that insulin resistance indirectly affects BMD, and a recent study reported that insulin resistance was inversely associated with bone mass [11]. Moreover, hyperinsulinemia was related to the inhibition of cortical bone development as assessed using peripheral quantitative computed tomography (pQCT) in adolescents [12]. Additionally, insulin resistance was associated with a smaller bone size and increased volumetric bone mineral density in non-diabetic postmenopausal women [13]. However, few studies have evaluated the relationship between insulin resistance and peak bone mass.

The present study evaluated the relationship between insulin resistance and the BMD of the femur and lumbar spine in Korean young adults aged 25–35 years, who are expected to exhibit near peak bone mass. We also investigated whether insulin resistance was an independent determinant for the femur and lumbar spine BMD after adjusting for possible confounding factors.

## Methods

### Subjects

Data from the Korean National Health and Nutrition Examination Survey (KNHANES) 2008–2010 were analyzed in the present study. The KNHANES was designed using a multistage and stratified sampling method, and it is a cross-sectional, nationally representative survey. The survey consists of three parts: a health interview survey, a health examination survey, and a nutritional survey. The Division of Chronic Disease Surveillance at the Korea Centers for Disease Control and Prevention conducts the survey annually [14], and additional details about the study design and method are provided elsewhere [15]. A total of 4,012 subjects of the randomly selected 29,235 participants in the KNHANES 2008–2010 were men and women aged 25−35 years. Of these subjects, 2,853 males and females completed dual-energy X-ray absorptiometry (DXA). Subjects with incomplete analytical data, including height, weight, BMI, blood pressure (BP), glucose, and insulin, were excluded (*n* = 72). Subjects who were currently receiving medication for previously diagnosed T2DM, which can influence insulin resistance, were also excluded (*n* = 31). A total of 2,750 Korean young adults (1,208 males and 1,542 females) were included in this study. The database is available to the public at the KNHANES website (http://knhanes.cdc.go.kr) [13], and all participants of the KNHANES provided informed consent.

### Measurements

A trained expert performed all anthropometric assessments according to standard methods. Briefly, height and body weight were assessed to the nearest 0.1 cm and 0.1 kg, respectively. Waist circumference (WC) was measured at the midline between the lower rib margin and iliac crest to the nearest 0.1 cm. The BMI was calculated as follows: weight (kg)/square of height (m^2^). The systolic blood pressure (SBP, mmHg) and diastolic blood pressure (DBP, mmHg) were measured three times on the right upper arm using a calibrated sphygmomanometer and an appropriately sized cuff. Each measurement was taken 2 min apart. The mean of the two last values was used for analysis.

Blood and urine samples were collected annually after the participants had fasted for ≥10 hours. Samples were immediately processed, refrigerated, and transported to a central laboratory (NeoDin Medical Institute, Seoul, Korea) for analysis within 24 hours. Routine biochemistry tests, including the levels of total cholesterol (T-C), triglycerides (TG), high-density lipoprotein cholesterol (HDL-C), and glucose, were measured enzymatically using an automatic Hitachi 7600 analyzer (Hitachi, Tokyo, Japan). The level of low-density lipoprotein cholesterol (LDL cholesterol) was calculated using the Friedewald’s equation:

$$\mathrm{LDL}\text{-}C\;(\mathrm{mg}/\mathrm{dL})=T\text{-}C\;(\mathrm{mg}/\mathrm{dL})\text{-}\;\mathrm{HDL}\text{-}C\;(\mathrm{mg}/\mathrm{dL})\;\text{-}\;\frac{\mathrm{TG}\;(\mathrm{mg}/\mathrm{dL})}{5}$$

The serum insulin and vitamin D levels were determined using an immunoradiometric assay and a 1470 Wizard Gamma Counter (Perkin-Elmer, Turku, Finland). The intra-assay coefficients of variation (CV) and inter-assay CV were 3.3%-4.6% and 4.8%-5.9% for insulin and 8.2%-11.0% and 8.6–12.5% for vitamin D, respectively.

DXA was conducted using a QDR Discovery fan beam densitometer (Hologic, Inc., Bedford, MA, USA). Well-trained and qualified technicians performed standardized daily quality control of DXA instruments using a spine phantom. Accurate and reliable data were generated and analyzed using industry standard techniques at the Korean Society of Osteoporosis. The DXA results were analyzed using the Hologic Discovery software (version 13.1). Examinations that revealed the presence of items that could affect the accuracy of the DXA results, such as prosthetic devices, implants, or other extraneous objects, were recorded as missing in the datasets for the regional and global DXA results. The KNHANES provided all DXA data, including bone mineral content (BMC, g), BMD (g/cm^2^), fat mass (g), lean mass, and fat percentage (fat mass/total mass × 100), according to individualized demographic information. The current study used the BMD of the total hip, femoral neck, femoral trochanter, femoral intertrochanter, lumbar spine, and whole body fat mass percentage in the analyses. The CV of the BMD measurement of the total hip, femoral neck, femoral trochanter, femoral intertrochanter and lumbar spine were less than 1.8%, 2.5%, 2.6%, 2.2% and 1.9%, respectively.

### Collection of general characteristic data

Smoking, alcohol intake, and physical activity were included in this study as lifestyle-related parameters. Smokers were defined as individuals who currently smoked or who had smoked ≥5 packs in their lifetime. Alcohol intake was defined as drinking ≥2 alcoholic beverages/month during the previous year. Physical activity was defined as meeting ≥1 of the following 3 criteria: (i) intense physical activity for 20 min for ≥3 days/week, (ii) moderate physical activity for 30 min for ≥5 days/week, or (iii) walking for 30 min for ≥5 days/week. Smoking, alcohol intake and physical activity were divided into “yes” or “no” groups. Education and household income were included as socio-demographic characteristics. Household income was reported in quartiles and categorized into the lowest quartile or ≥ the second quartile group. The education level was divided into the following two groups: ≤ high school or ≥ university. An assessment of gynecological history characteristics was included in the general characteristics of females. The gynecological characteristics included labor, the use of oral contraceptives and age at menarche. Labor was divided into two groups: childbirth ≥1 in a lifetime or never. The use of oral contraceptives was also categorized into two groups: the administration of oral contraceptives ≥1 in a lifetime or never. The age at menarche was defined as the age of the first menstrual period. This information was obtained using an open-ended questionnaire: “At what age did your first menstrual period begin?” Participants were divided into the following three categories according to age at menarche: early (<12 years), normal [12–16], or late (≥16 years) age at menarche [17]. The general participant characteristics were obtained from the KNHANES.

### Definition of insulin resistance indices

Insulin resistance was determined using the homeostasis model assessment for insulin resistance (HOMA-IR) [18] and the serum fasting insulin level. The HOMA-IR was calculated using the following equation:

$$\mathrm{HOMA}\text{-}\mathrm{IR}=\frac{\mathrm{fasting}\;\mathrm{insulin}\;(\mathrm{\mu U}/\mathrm{mL})\times \mathrm{fasting}\;\mathrm{glucose}\;(\mathrm{mmol}/L)}{22.5}$$

### Statistical analyses

All analyses were performed using the SPSS software for Windows (SPSS version 22.0, IBM SPSS Inc., Chicago, IL, USA). The participant characteristics are presented according to the gender-specific quartiles of the HOMA-IR and serum fasting insulin. Normally distributed variables are presented as the means ± standard error (SE), and categorical variables are presented as a percentage (%). Differences in categorical variables and normally distributed variables were analyzed using the chi-squared test and an analysis of variance (ANOVA), respectively. An analysis of covariance (ANCOVA) was used to evaluate the relationship of the HOMA-IR and serum fasting insulin with the BMD of the total hip, femoral neck, femoral trochanter, femoral intertrochanter, and lumbar spine after adjusting for independent factors, such as age, height, weight, whole body fat mass percentage, SBP, DBP, T-C, TG, HDL-C, LDL-C, vitamin D, smoking, alcohol intake, physical activity, education level, and household income in both genders, as well as labor, the use of OCS and age at menarche in females according to the gender-specific quartiles of HOMA-IR and serum fasting insulin. Unadjusted and adjusted associations between the BMD as dependent variables and HOMA-IR or serum fasting insulin as independent variables were assessed using univariate linear regression analyses before adjustments and multivariate linear regression analyses after adjustments among all study participants. In model 1, univariate linear regression analyses were conducted with no adjustments. The multivariate linear regression analyses were performed after adjusting for gender, age, height, weight, and the whole body fat mass percentage among all subjects in model 2. In model 3, the multivariate linear regression analyses of the BMD as the dependent variable and HOMA-IR and serum fasting insulin level as independent variables were conducted after controlling for confounding factors, such as gender, age, BMI, whole body fat mass percentage, SBP, DBP, T-C, TG, HDL-C, LDL-C, vitamin D, smoking, alcohol intake, physical activity, education level, and household income in both genders as well as labor, use of oral contraceptives (OCs), and age at menarche in females. The corresponding standardized regression coefficient (β), standard error (SE), and coefficient of determination (R^2^) were determined. The subgroup analyses of the unadjusted and adjusted associations according to the respective model were also conducted in males and females, although the interaction terms between the insulin resistance indices and gender were not observed in our analyses. The corresponding standardized β, SE, and R^2^ were calculated in subgroup analyses. All significance differences were determined using a two-tailed method, and a *P* value <0.05 was considered statistically significant.

## Results

### Clinical characteristics of the study population according to the gender-specific quartiles of the HOMA-IR (Table 1) and serum fasting insulin levels (S1 Table)

The HOMA-IR and serum insulin level significantly differed between male and female participants. The respective 25th, median, and 75th percentile of the HOMA-IR and serum fasting insulin level were 1.60 and 7.43 μU/mL, 2.05 and 9.22 μU/mL, and 2.73 and 12.04 μU/mL in males and 1.50 and 7.04 μU/mL, 1.90 and 8.74 μU/mL, and 2.42 and 10.87 μU/mL in females (*P*<0.001). **Table 1** shows the clinical characteristics of the study participants. Both male and female subjects in the Q4 of HOMA-IR tended to exhibit increased weight, WC, BMI SBP, DBP, glucose levels, insulin levels, T-C levels, TG levels, and HDL-C levels (*P*<0.001). Male participants in the Q4 of HOMA-IR were also more likely to exhibit a higher BMD for the total hip (*P* = 0.003), femoral trochanter (*P* = 0.001), and whole body fat percentage (*P*<0.001), whereas females exhibited a higher BMD of the total femur (*P*<0.001), femoral neck (*P*<0.013), femoral trochanter (*P*<0.001), femoral intertrochanter (*P*<0.001), lumbar spine (*P*<0.001), and whole body fat percentage (*P*<0.001). Females in the Q4 of HOMA-IR tended to give birth during their lifetime (*P*<0.001). The clinical characteristics according to the gender-specific quartiles of the serum fasting insulin level were similar to HOMA-IR (**S1 Table**).

**Table 1:** Characteristics of study participants according to the gender-specific homoeostasis model assessment-estimated insulin resistance (HOMA-IR) quartiles in Korean males (n = 1,208) and females (*n* = 1,542).

	HOMA-IR				*P*
	Q1	Q2	Q3	Q4	
Males	≤1.60	1.60–2.05	2.05–2.73	>2.73	
	*n* = 302	*n* = 302	*n* = 302	*n* = 302	
Age (yr)	30.20 ± 0.19	30.41 ± 0.19	30.03 ± 0.19	30.41 ± 0.18	0.741
Height (cm)	174.03 ± 0.33	173.59 ± 0.30	174.04 ± 0.32	174.34 ± 0.35	0.346
Weight (kg)	66.92 ± 0.57	70.99 ± 0.52	74.70 ± 0.57	80.34 ± 0.73	<0.001
WC (cm)	77.75 ± 0.46	81.28 ± 0.43	84.33 ± 0.46	89.26 ± 0.55	<0.001
BMI (kg/m^2^)	22.07 ± 0.17	23.54 ± 0.16	24.65 ± 0.17	26.40 ± 0.21	<0.001
SBP (mmHg)	110.30 ± 0.64	112.86 ± 0.59	113.59 ± 0.65	115.21 ± 0.75	<0.001
DBP (mmHg)	72.94 ± 0.54	75.60 ± 0.55	75.99 ± 0.58	77.67 ± 0.61	<0.001
Glucose (mg/dL)	86.01 ± 0.37	88.89 ± 0.36	91.75 ± 0.45	95.32 ± 0.50	<0.001
Insulin (μU/mL)	6.20 ± 0.06	8.33 ± 0.04	10.42 ± 0.07	16.88 ± 0.43	<0.001
T-C (mg/dL)	176.48 ± 1.93	181.33 ± 1.88	183.70 ± 1.87	192.49 ± 2.05	<0.001
TG (mg/dL)	113.80 ± 6.50	127.28 ± 4.39	142.60 ± 5.83	198.96 ± 8.20	<0.001
HDL-C (mg/dL)	53.72 ± 0.68	51.21 ± 0.66	48.65 ± 0.58	45.57 ± 0.57	<0.001
LDL-C (mg/dL)	100.01 ± 1.94	104.67 ± 1.72	106.53 ± 1.77	107.13 ± 1.93	0.005
Vitamin D (ng/mL)	18.62 ± 0.39	17.94 ± 0.35	17.99 ± 0.34	17.96 ± 0.37	0.237
BMD (g/cm^2^)					
Total hip	0.99 ± 0.01	1.00 ± 0.01	1.01 ± 0.01	1.02 ± 0.01	0.003
Femoral neck	0.69 ± 0.01	0.69 ± 0.01	0.69 ± 0.01	0.70 ± 0.01	0.150
Femoral trochanter	1.19 ± 0.01	1.20 ± 0.01	1.21 ± 0.01	1.22 ± 0.01	0.001
Femoral intertrochanter	0.88 ± 0.01	0.88 ± 0.01	0.89 ± 0.01	0.89 ± 0.01	0.265
Lumbar spine	0.99 ± 0.01	1.00 ± 0.01	1.00 ± 0.11	1.00 ± 0.01	0.151
Whole body fat percentage	18.33 ± 0.33	21.40 ± 0.34	23.22 ± 0.31	25.02 ± 0.29	<0.001
Smoking (%)	224 (74.2%)	223 (73.8%)	222 (73.5%)	227 (75.2%)	0.971
Alcohol intake (%)	252 (83.4%)	232 (76.8%)	253 (83.8%)	246 (81.5%)	0.105
Physical activity (%)	183 (60.6%)	172 (56.9%)	188 (62.3%)	160 (53.0%)	0.097
Education ≤ high school (%)	130 (43.1%)	132 (43.7%)	116 (38.4%)	115 (38.1%)	0.344
House income ≤1st quartiles (%)	28 (9.3%)	22 (7.3%)	24 (7.9%)	20 (6.6%)	0.656
Females	≤1.50	1.50–1.90	1.90–2.42	>2.42	
	*n* = 385	*n* = 386	*n* = 386	*n* = 385	
Age (yr)	30.70 ± 0.16	30.70 ± 0.16	30.66 ± 0.16	30.67 ± 0.16	0.872
Height (cm)	160.62 ± 0.29	160.46 ± 0.28	160.75 ± 0.29	160.78 ± 0.27	0.533
Weight (kg)	52.87 ± 0.32	54.23 ± 0.39	55.86 ± 0.41	62.73 ± 0.59	<0.001
WC (cm)	70.34 ± 0.35	71.83 ± 0.38	73.36 ± 0.42	79.40 ± 0.55	<0.001
BMI (kg/m^2^)	20.50 ± 0.12	21.05 ± 0.13	21.63 ± 0.15	24.24 ± 0.21	<0.001
SBP (mmHg)	101.03 ± 0.48	101.71 ± 0.52	103.01 ± 0.47	106.96 ± 0.58	<0.001
DBP (mmHg)	66.35 ± 0.42	66.65 ± 0.46	67.63 ± 0.42	70.51 ± 0.44	<0.001
Glucose (mg/dL)	83.72 ± 0.29	87.11 ± 0.30	89.42 ± 0.31	93.74 ± 0.43	<0.001
Insulin (μU/mL)	6.10 ± 0.05	7.92 ± 0.04	9.71 ± 0.04	14.36 ± 0.27	<0.001
T-C (mg/dL)	169.14 ± 1.37	166.02 ± 1.49	171.81 ± 1.31	179.39 ± 1.60	<0.001
TG (mg/dL)	65.92 ± 1.95	74.38 ± 1.95	91.97 ± 3.90	108.65 ± 3.35	<0.001
HDL-C (mg/dL)	60.82 ± 0.61	58.52 ± 0.60	57.61 ± 0.63	54.19 ± 0.64	<0.001
LDL-C (mg/dL)	95.14 ± 1.22	92.62 ± 1.28	95.81 ± 1.24	103.47 ± 1.47	<0.001
Vitamin D (ng/mL)	16.81 ± 0.29	16.84 ± 0.32	16.29 ± 0.28	16.37 ± 0.29	0.157
BMD (g/cm^2^)					
Total hip	0.87 ± 0.01	0.88 ± 0.01	0.88 ± 0.01	0.91 ± 0.01	<0.001
Femoral neck	0.63 ± 0.01	0.64 ± 0.00	0.63 ± 0.01	0.65 ± 0.01	<0.001
Femoral trochanter	1.04 ± 0.00	1.06 ± 0.00	1.06 ± 0.00	1.09 ± 0.00	<0.001
Femoral intertrochanter	0.75 ± 0.01	0.76 ± 0.01	0.76 ± 0.01	0.78 ± 0.01	<0.001
Lumbar spine	0.96 ± 0.01	0.98 ± 0.01	0.97 ± 0.01	1.01 ± 0.01	<0.001
Whole body fat percentage	29.37 ± 0.27	30.75 ± 0.27	31.48 ± 0.27	34.21 ± 0.27	<0.001
Smoking (%)	55 (14.3%)	54 (14.0%)	56 (14.5%)	63 (16.4%)	0.788
Alcohol intake (%)	191 (49.6%)	190 (49.2%)	193 (50.0%)	205 (53.3%)	0.665
Physical activity (%)	195 (50.7%)	174 (45.1%)	175 (45.3%)	194 (50.4%)	0.224
Education ≤high school (%)	145 (37.7%)	137 (35.5%)	158 (40.9%)	196 (50.9%)	<0.001
House income ≤1st quartiles (%)	20 (5.2%)	17 (4.4%)	25 (6.5%)	23 (6.0%)	0.608
Labor ≥1/lifetime (%)	49 (12.7%)	90 (23.3%)	91 (23.6%)	90 (23.4%)	<0.001
Age at menarche <12 yr (%)	30 (7.8%)	34 (8.8%)	32 (8.3%)	40 (10.4%)	0.611
Use of OCs (%)	8 (2.1%)	5 (1.3%)	10 (2.6%)	6 (1.6%)	0.558

Data are presented as the means ± SE (standard error).

HOMA-IR, homeostatic model assessment of insulin resistance; WC, waist circumference; BMI, body mass index; SBP, systolic blood pressure; DBP, diastolic blood pressure; T-C, total cholesterol; TG, triglyceride; HDL-C, high-density lipoprotein cholesterol; LDL-C, low-density lipoprotein cholesterol; BMD, bone mineral density; OCs, oral contraceptives.

### Adjusted means for the BMD of the femur and lumbar spine according to the gender-specific quartiles of the HOMA-IR (Fig 1) and serum fasting insulin levels (S1 Fig)

**Fig 1:**
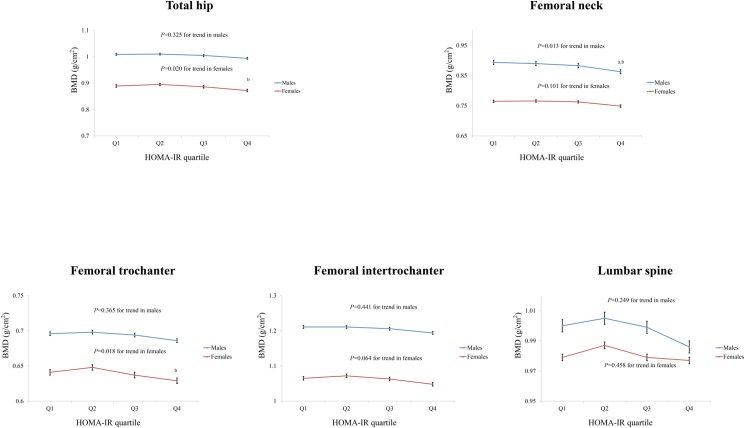
Adjusted means for the bone mineral density (BMD) of the total hip, femoral neck, femoral trochanter, femoral intertrochanter, and lumbar spine according to the HOMA-IR quartiles in Korean males (*n* = 1,208) and females (*n* = 1,542). The BMD values of the total hip, femoral neck, femoral trochanter, femoral intertrochanter, and lumbar spine were adjusted for age, height, weight, whole body fat percentage, systolic blood pressure (SBP), diastolic blood pressure (DBP), total cholesterol, triglyceride, high-density lipoprotein cholesterol (HDL-C), low-density lipoprotein cholesterol (LDL-C), vitamin D, smoking, alcohol intake, physical activity, education level, and household income in both genders as well as labor, the use of oral contraceptives (OCs), and age at menarche in females using an analysis of covariance (ANCOVA) according to the gender-specific HOMA-IR quartile. ^a^; *P*<0.05, vs. first quartile, ^b^; *P*<0.05, vs. second quartile, ^c^; *P*<0.05, vs. third quartile. BMD, bone mineral density; HOMA-IR, homeostatic model assessment of insulin resistance.

The adjusted means for the BMD of the total hip, femoral neck, femoral trochanter, femoral intertrochanter, and lumbar spine according to the gender-specific quartiles of the HOMA-IR were calculated using ANCOVA after adjusting for possible confounding factors. **Fig 1** shows the adjusted means for the BMD according to the gender-specific quartiles of the HOMA-IR. The quartiles of the HOMA-IR were significantly and inversely correlated with the BMD of the total hip in females (*P* = 0.020 for the trend). The total hip BMD was significantly lower in the Q4 of the HOMA-IR than the Q2 (*P* = 0.012) of the HOMA-IR in females. A significant inverse association was observed between the quartiles of the HOMA-IR and femoral neck BMD (*P* = 0.013 for the trend) in males. In males, the femoral neck BMD in the highest HOMA-IR quartile was lower than that in the Q1 (*P* = 0.018) or Q2 (*P* = 0.030) of the HOMA-IR. Moreover, the HOMA-IR quartiles significantly and inversely correlated with the BMD of the femoral trochanter in females (*P* = 0.018 for the trend). The femoral trochanter BMD was significantly lower in the Q4 of the HOMA-IR than the Q2 of the HOMA-IR in females (*P* = 0.012). A marginal inverse association was found between the HOMA-IR quartiles and femoral intertrochanter BMD (*P* = 0.064 for trend) in females. In females, the femoral intertrochanter BMD was lower in the Q4 of the HOMA-IR than the Q2 of the HOMA-IR (*P* = 0.047). **S1 Fig** shows the adjusted means for the BMD according to the gender-specific quartiles of the serum fasting insulin level. A significant inverse association was found between the serum fasting insulin quartiles and BMD of the femoral neck (*P* = 0.003 for the trend) as well as the lumbar spine (*P* = 0.029 for the trend) in males and between the serum fasting insulin quartiles and BMD of the total hip (*P* = 0.001 for the trend), femoral neck (*P* = 0.009 for the trend), femoral trochanter (*P* = 0.005 for the trend), and femoral intertrochanter (*P* = 0.004 for the trend) in females.

### Unadjusted and adjusted associations of the BMD of the femur and lumbar spine with the HOMA-IR (Table 2) and serum fasting insulin (S2 Table)

The associations between the BMD of the femur and lumbar spine as well as insulin resistance were assessed using a univariate linear regression analysis before adjustment and a multivariate linear regression analysis after adjustment. The unadjusted and adjusted associations are presented in **Table 2**. In the univariate linear regression analyses of all subjects, the HOMA-IR and serum fasting insulin were significantly positively associated with weight (β = 0.365, *P*<0.001 and β = 0.354, *P*<0.001), BMI (β = 0.395, *P*<0.001 and β = 0.388, *P*<0.001), and whole body fat percentage (β = 0.162, *P*<0.001 and β = 0.108, *P*<0.001). The univariate linear regression analyses identified a significant positive association between the HOMA-IR and BMD of the total hip, femoral neck, femoral trochanter, femoral intertrochanter, and lumbar spine in the total study population (*P*<0.001). In model 2, after adjusting for gender, age, height, weight, and the whole body fat mass percentage, a significant inverse association was found between the HOMA-IR and BMD of the total hip (β = −0.063, *P*<0.001), BMD of the femoral neck (β = −0.086, *P*<0.001), BMD of the femoral trochanter (β = −0.064, *P*<0.001), BMD of the femoral intertrochanter (β = −0.051, *P* = 0.002), and BMD of the lumbar spine (β = −0.073, *P*<0.001) in all study participants. In model 3, the HOMA-IR was significantly, independently and inversely associated with the BMD of the total hip (β = −0.052, *P* = 0.002), femoral neck (β = −0.072, *P*<0.001), femoral trochanter (β = −0.055, *P* = 0.003), femoral intertrochanter (β = −0.041, *P* = 0.015), and lumbar spine (β = −0.063, *P* = 0.001) in all study participants. In the subgroup analyses of model 3, significant inverse associations were observed between the HOMA-IR and the BMD of the total hip in males (β = −0.065, *P* = 0.022), femoral neck in males (β = −0.100, *P*<0.001), femoral trochanter in males (β = −0.066, *P* = 0.024), and lumbar spine in males (β = −0.084, *P* = 0.005), but a significant inverse association was identified between the HOMA-IR and BMD of the femoral neck in females (β = −0.051, *P* = 0.048) using a multivariate linear regression analysis. Marginal inverse associations were found between the HOMA-IR and BMD of the total hip in females (β = −0.044, *P* = 0.079) and in the femoral intertrochanter in males (β = −0.054, *P* = 0.066) in subgroup analyses of model 3.

**Table 2:** Unadjusted and adjusted associations between the homeostatic model assessment of insulin resistance (HOMA-IR) and bone mineral density (BMD) of the total hip, femoral neck, femoral trochanter, femoral intertrochanter, and lumbar spine in Korean males and females (*n* = 2,750).

	HOMA-IR											
	Model 1				Model 2				Model 3			
	β	SE	R^2^	*P*	β	SE	R^2^	*P*	β	SE	R^2^	*P*
Total hip (g/cm^2^)												
All (*n* = 2,750)	0.136	0.002	0.019	<0.001	−0.063	0.002	0.388	<0.001	−0.052	0.002	0.397	0.002
Males (*n* = 1,208)	0.068	0.0025	0.005	0.018	−0.080	0.002	0.224	0.005	−0.065	0.002	0.237	0.022
Females (*n* = 1,542)	0.135	0.003	0.018	<0.001	−0.051	0.002	0.226	0.039	−0.044	0.003	0.239	0.079
Femoral neck (g/cm^2^)												
All (*n* = 2,750)	0.096	0.002	0.009	<0.001	−0.086	0.002	0.375	<0.001	−0.072	0.002	0.383	<0.001
Males (*n* = 1,208)	0.020	0.003	0.000	0.488	−0.118	0.002	0.240	<0.001	−0.100	0.002	0.256	<0.001
Females (*n* = 1,542)	0.095	0.003	0.009	<0.001	−0.062	0.003	0.172	0.015	−0.051	0.003	0.179	0.048
Femoral trochanter (g/cm^2^)												
All (*n* = 2,750)	0.103	0.001	0.011	<0.001	−0.064	0.001	0.244	<0.001	−0.055	0.001	0.257	0.003
Males (*n* = 1,208)	0.029	0.002	0.001	0.319	−0.081	0.002	0.176	0.005	−0.066	0.002	0.189	0.024
Females (*n* = 1,542)	0.130	0.002	0.017	<0.001	−0.038	0.002	0.197	0.134	−0.039	0.002	0.212	0.129
Femoral intertrochanter (g/cm^2^)												
All (*n* = 2,750)	0.143	0.002	0.020	<0.001	−0.051	0.002	0.369	0.002	−0.041	0.002	0.377	0.015
Males (*n* = 1,208)	0.081	0.003	0.007	0.005	−0.066	0.003	0.193	0.021	−0.054	0.003	0.205	0.066
Females (*n* = 1,542)	0.137	0.003	0.019	<0.001	−0.039	0.003	0.207	0.118	−0.031	0.003	0.216	0.219
Lumbar spine (g/cm^2^)												
All (*n* = 2,750)	0.075	0.002	0.006	<0.001	−0.073	0.002	0.153	<0.001	−0.063	0.002	0.165	0.001
Males (*n* = 1,208)	0.017	0.002	0.000	0.544	−0.097	0.002	0.143	0.001	−0.084	0.002	0.155	0.005
Females (*n* = 1,542)	0.125	0.003	0.016	<0.001	−0.036	0.003	0.184	0.154	−0.035	0.003	0.201	0.174

Model 1: Univariate linear regression analyses between the homeostatic model assessments of insulin resistance (HOMA-IR) and bone mineral density (BMD) of the total hip, femur neck, femur trochanter, femur intertrochanter, and lumbar spine were conducted with no adjustments.

Model 2: Multivariate linear regression analyses between the HOMA-IR and BMD of the total hip, femur neck, femur trochanter, femur intertrochanter, and lumbar spine were conducted after adjusting for gender, age, height, weight, and the whole body fat mass percentage.

Model 3: Multivariate linear regression analyses between the HOMA-IR and BMD of the total hip, femur neck, femur trochanter, femur intertrochanter, and lumbar spine were conducted after adjusting for gender, age, height, weight, whole body fat mass percentage, systolic blood pressure (SBP), diastolic blood pressure (DBP), total cholesterol, triglyceride, high-density lipoprotein cholesterol (HDL-C), low-density lipoprotein cholesterol (LDL-C), vitamin D, smoking, alcohol intake, physical activity, education level, and household income in both genders as well as labor, use of oral contraceptives (OCs), and age at menarche in females.

Subgroup analyses were conducted after adjusting for previously described confounding factors according to gender using a multivariate linear regression analysis.

β, standardized regression coefficient; SE, standard error; R2, coefficient of determination.

**S2 Table** shows the unadjusted and adjusted associations between the BMD of the femur and lumbar spine and the serum fasting insulin level. In model 3, the serum fasting insulin level was significantly, independently and inversely associated with the BMD of the total hip (β = −0.055, *P* = 0.001), femoral neck (β = −0.072, *P*<0.001), femoral trochanter (β = −0.055, *P* = 0.003), femoral intertrochanter (β = −0.045, *P* = 0.009), and lumbar spine (β = −0.064, *P* = 0.001) in all study subjects according to a multivariate linear regression analysis. In the subgroup analyses of model 3, significant inverse associations were also observed between the serum fasting insulin level and BMD of the total hip in males (β = −0.068, *P* = 0.017), femoral neck in males (β = −0.102, *P*<0.001), femoral trochanter in males (β = −0.070, *P* = 0.018), and lumbar spine in males (β = −0.092, *P* = 0.002). Marginal inverse associations were found between the serum fasting insulin level and BMD of the total hip in females (β = −0.047, *P* = 0.061), femoral neck in females (β = −0.048, *P* = 0.062), and femoral intertrochanter in males (β = −0.056, *P* = 0.054) in the subgroup analyses of model 3.

## Discussion

Data from this nationwide cross-sectional study revealed an independent inverse association between the insulin resistance, which was presented as the HOMA-IR and serum insulin level, and the BMD of the total hip, femoral neck, femoral trochanter, femoral intertrochanter, and lumbar spine in 2,750 Korean males and females aged 25–35 years, who are expected to exhibit near peak bone mass after adjusting for possible confounders in a multivariate linear regression analysis. However, these inverse associations of the HOMA-IR and serum fasting insulin with the BMD of the femur and lumbar spine were significant in the subgroup analyses of males, whereas these associations were attenuated in the subgroup analyses of females after adjusting for possible confounders using a multivariate linear regression analysis.

Insulin resistance or hyperinsulinemia may be related to bone mass because insulin plays an important role in the anabolic effects on bone mass [19]. Specifically, hyperinsulinemia may negatively impact the binding of sex hormones with sex hormone binding globulin (SHBG) and consequently be related to increases in free sex hormone levels, which play a role in higher bone mass [10]. Hyperinsulinemia is positively associated with bone mass [7,20], and individuals with T2DM of both genders exhibit a higher BMD [9]. Moreover, men and women with obesity, which is closely related to insulin resistance, exhibited higher BMD values in previous studies [20]. Patients with Berardinelli-Seip Congenital Lipodystrophy (BSCL), which is a rare autosomal recessive syndrome characterized by a difficulty storing lipid in adipocytes, low body fat, hypertriglyceridemia, and fatty liver, exhibited a higher HOMA-IR and higher BMD [21]. However, reports of the relationship between insulin resistance and bone mass have been inconsistent. Some studies found that insulin resistance was positively associated with bone mass, but this relationship was attenuated by or not significant after adjusting for weight and BMI [10,22]. Recent Korean studies demonstrated an inverse association between insulin resistance and bone mass in men aged ≥20 years [11], whereas other studies reported that insulin resistance was related to a lower BMD in adolescents [23] and patients with T2DM [24]. A recent study also demonstrated that insulin resistance is inversely associated with trabecular and cortical bone size in non-diabetic men at the age of peak bone mass [25]. An inverse relationship between insulin resistance and composite indices of femoral neck strength was also suggested [26,27]. The present study observed an inverse association between the insulin resistance indices (HOMA-IR and serum insulin level) and near peak BMD of the total hip, femoral neck, femoral trochanter, femoral intertrochanter, and lumbar spine in the entire study population of young Korean adults after adjusting for possible confounding factors using a multivariate linear regression analysis. Our results are consistent with a previous study that suggested an inverse association between insulin resistance and BMD.

Several possible explanations for the inverse association of insulin resistance with bone mass may be proposed. For example, insulin resistance may directly affect bone mass because bone is a metabolically active organ and a target organ of insulin [28,29]. Moreover, insulin receptor signaling in osteoblasts is important for the development of osteoblasts [29], and insulin signals in osteoblasts activate osteocalcin production, which regulates insulin sensitivity and promotes glucose metabolism [30]. One animal study demonstrated that insulin resistance induced by a 12-week high fat diet (HFD) impaired osteoblastic insulin signaling, osteoblast proliferation, and osteoblast survival and resulted in osteoporosis of the jawbone in a mouse model [31]. Another animal study also demonstrated that a HFD induced insulin resistance in the bones of mice, and insulin resistance in osteoblasts contributed to the development of systematic insulin resistance in a mouse model of T2DM [32]. These animal studies suggest that bone is a site of insulin resistance, and the interruption of osteoblastic insulin signaling in insulin-resistant subjects may lead to a reduction in bone mass. However, reports of the relationship between insulin resistance and bone metabolism from animal studies have been inconsistent. For example, fibroblast growth factor 21 (FGF21) promotes insulin sensitivity but causes bone loss, and the insulin-like growth factor binding protein 1 (IGFBP-1) plays a role in the liver-bone hormonal linkage that promotes osteoclastogenesis and bone resorption, and it is an essential mediator of FGF21-induced bone loss [33]. The current study could not further investigate this hypothesis because the KNHANES does not provide information regarding the biochemical markers of bone formation and resorption or insulin signaling in human osteoblasts. Thus, further studies are needed to evaluate this relationship.

Indirect factors may also contribute to insulin resistance and the changes in bone mass. For example, increased fat mass, which is related to insulin resistance, may also affect BMD. In a study on non-diabetic women, a significant inverse association between the bone marrow adiposity and BMD were observed [34]. A high lean mass and low fat mass exert protective effects on bone health, and a higher fat mass may be related to the detrimental effects on BMD [11]. Moreover, fat mass was inversely related to BMC after the removal of the mechanical loading effect in males and females [35]. In the current study, the whole-body fat mass was controlled as a confounding factor in the current study. Increases in the levels of pro-inflammatory cytokines, such as interleukin-6 (IL-6) and tumor necrosis factor α (TNF-α), are observed in individuals with insulin-resistance, which may induce bone loss by stimulating osteoclast activity [36,37]. Furthermore, altered lipid levels in insulin-resistant subjects may also be associated with the BMD. Specifically, an inverse relationship was observed between the lumbar spine and the whole-body BMD and between the serum T-C and LDL levels [38], and an atherogenic lipid profile, defined as T-C ≥240 mg/dL, LDL-C ≥160 mg/dL or lipoprotein-a ≥25 mg/dL, has been associated with a lower lumbar and femoral BMD [39]. Notably, a significant inverse association was maintained between insulin resistance and the near peak BMD of the femur and lumbar spine in Korean individuals aged 25–35 years after adjusting for possible confounding factors, including fat mass, which was assessed in our study based on the whole body fat mass percentage and lipid profiles using a multivariate linear regression analysis. However, pro-inflammatory cytokines could not be adjusted as confounders in our study.

Unexpectedly, the subgroup analysis in females identified a marginally significant inverse association between insulin resistance indices and the near peak bone mass of the total hip and femoral neck (significant association of the HOMA-IR and BMD of the femoral neck), but no significant inverse relationship was found between insulin resistance indices and the near peak bone mass of the femoral trochanter, femoral intertrochanter, or the lumbar spine. In males, the subgroup analysis identified a significant inverse relationship between the insulin resistance indices and BMD of the femur and lumbar spine (marginal association between the HOMA-IR and serum fasting insulin and femoral intertrochanter). This result may be related to differences in gender that influence the peak bone mass [40]. Sex hormones (estradiol) are related to the differences in bone mass accrual between genders, but genetic factors play a significant role in the gains of the peak bone mass, which accounts for approximately 60–80% of its variance [41]. The rate and magnitude of bone mass gain during the pubertal years may markedly differ among skeletal sites and individuals [42]. However, the data were adjusted for labor, use of OCS, and age at menarche in females in our study, which may be related to sex hormones that influence peak bone mass. Nevertheless, the data could not be adjusted for the measured estradiol levels. Alternatively, the definition of insulin resistance, which was assessed by the HOMA-IR and serum fasting insulin levels in our study, may be related to the results of the current study. This study showed a significant association between the HOMA-IR and BMD of the femoral neck in females, but a marginally significant association between the serum insulin and BMD of the femoral neck in females.

Our study was subject to potential limitations. First, this study was designed as a cross-sectional study, and the exact causality could not be determined. However, possible explanations underlying the relationship between insulin resistance and BMD, including direct and indirect mechanisms, have been previously described. Second, lifestyle-related and dietary status data were collected using self-reporting methods in the KNHANES; thus, we could not utilize objectively measured data in our study. This collection may have affected the level of accuracy of the data, potentially leading to recall and social desirability biases. However, this study was conducted on a nationwide basis, and lifestyle-related and dietary status were included as confounders in the ANCOVA and the multivariate linear regression analyses. Finally, the HOMA-IR and serum fasting insulin level were used as insulin resistance indices in the current study, but the gold standard test to determine insulin resistance is the hyperinsulinemic-euglycemic clamp study. However, insulin resistance could not be exactly analyzed because the participants in the KNHANES did not undergo the clamp test. Nevertheless, the HOMA-IR correlates well with the results of the clamp test [43], and the HOMA-IR is a simple, reliable, and reproducible surrogate measurement of insulin resistance in large epidemic studies [44]. Despite these potential limitations, the present study is notable because it examined a nationally representative large population, presented findings that differ from previous studies suggesting a positive association between resistance and BMD and supports recent reports showing that insulin resistance is independently and inversely associated with the BMD in animals and humans.

In conclusion, this nationally representative cross-sectional study demonstrated that insulin resistance, as assessed based on the HOMA-IR and serum fasting insulin levels, was significantly and independently inversely associated with the BMD of the total hip, femoral neck, femoral trochanter, femoral intertrochanter, and lumbar spine in all study participants aged 25–35 years, who are expected to exhibit near peak BMD values in a multivariate linear regression analysis after adjusting for possible confounding factors. Our results suggest that insulin resistance is independently and inversely related to the near peak BMD of the femur and lumbar spine.

## Supporting information

S1 Fig Adjusted means for the bone mineral density (BMD) of the total hip, femoral neck, femoral trochanter, femoral intertrochanter, and lumbar spine according to the serum fasting insulin quartiles in Korean males (*n* = 1,208) and females (*n* = 1,542). The BMD of the total hip, femoral neck, femoral trochanter, femoral intertrochanter, and lumbar spine were adjusted for age, height, weight, whole body fat percentage, systolic blood pressure (SBP), diastolic blood pressure (DBP), total cholesterol, triglyceride, high-density lipoprotein cholesterol (HDL-C), low-density lipoprotein cholesterol (LDL-C), vitamin D, smoking, alcohol intake, physical activity, education level, and household income in both genders as well as labor, the use of oral contraceptives (OCs), and age at menarche in females using an analysis of covariance (ANCOVA) according to the gender-specific serum fasting insulin quartiles. ^a^; *P*<0.05, vs. first quartile, ^b^; *P*<0.05, vs. second quartile, ^c^; *P*<0.05, vs. third quartile. BMD; bone mineral density.
(TIF)

S1 Table Characteristics of study participants according to the gender-specific serum insulin quartiles in Korean males (*n* = 1,208) and females (*n* = 1,542). (DOCX)

S2 Table Unadjusted and adjusted associations between the serum fasting insulin level and bone mineral density (BMD) of the total hip, femoral neck, femoral trochanter, femoral intertrochanter, and lumbar spine in Korean males and females (*n* = 2,750). (DOCX)
