# Supplementary figures and images for: Association of insulin resistance with near peak bone mass in the femur and lumbar spine of Korean adults aged 25-35: The Korean National Health and Nutrition Examination Survey 2008-2010
Source: PLoS One. 2017 Jul 13;12(7):e0177311. doi: 10.1371/journal.pone.0177311 (PMC5509105; doi:10.1371/journal.pone.0177311)

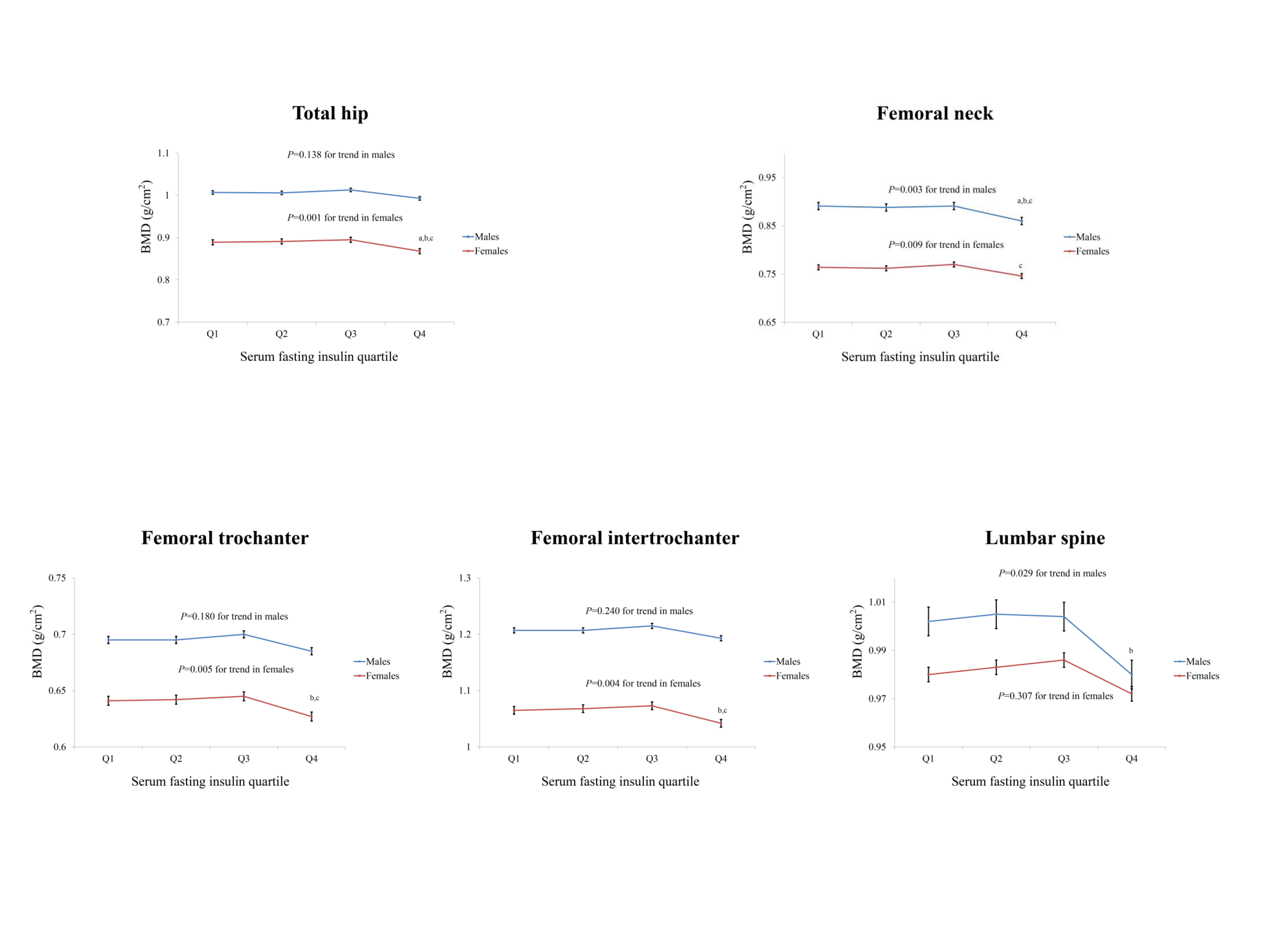

Supplement: S1 Fig — The BMD of the total hip, femoral neck, femoral trochanter, femoral intertrochanter, and lumbar spine were adjusted for age, height, weight, whole body fat percentage, systolic blood pressure (SBP), diastolic blood pressure (DBP), total cholesterol, triglyceride, high-density lipoprotein cholesterol (HDL-C), low-density lipoprotein cholesterol (LDL-C), vitamin D, smoking, alcohol intake, physical activity, education level, and household income in both genders as well as labor, the use of oral contraceptives (OCs), and age at menarche in females using an analysis of covariance (ANCOVA) according to the gender-specific serum fasting insulin quartiles. a; P<0.05, vs. first quartile, b; P<0.05, vs. second quartile, c; P<0.05, vs. third quartile. BMD; bone mineral density. (TIF) [file pone.0177311.s001.tif]
