# Supplementary material for: Association of insulin resistance with near peak bone mass in the femur and lumbar spine of Korean adults aged 25-35: The Korean National Health and Nutrition Examination Survey 2008-2010
Source: PLoS One. 2017 Jul 13;12(7):e0177311. doi: 10.1371/journal.pone.0177311 (PMC5509105; doi:10.1371/journal.pone.0177311)
Supplement: S1 Table — (DOCX) [file pone.0177311.s002.docx]

**S1 Table. Characteristics of study participants according to the gender-specific serum insulin quartiles in Korean males (*n*=1,208) and females (*n*=1,542).**

|  | Serum fasting insulin (μU/mL) | | | | *P* |
| --- | --- | --- | --- | --- | --- |
|  | Q1 | Q2 | Q3 | Q4 |  |
| Males | ≤7.43 | 7.43-9.22 | 9.22-12.04 | >12.04 |  |
|  | *n*=302 | *n*=302 | *n*=302 | *n*=302 |  |
| Age (yr) | 30.39 ± 0.19 | 30.36 ± 0.19 | 29.98 ± 0.19 | 30.32 ± 0.19 | 0.476 |
| Height (cm) | 173.84 ± 0.33 | 173.93 ± 0.30 | 173.95 ± 0.31 | 174.30 ± 0.35 | 0.339 |
| Weight (kg) | 66.74 ± 0.55 | 71.84 ± 0.56 | 74.13 ± 0.56 | 80.23 ± 0.73 | <0.001 |
| WC (cm) | 77.71 ± 0.46 | 82.09 ± 0.46 | 83.73 ± 0.46 | 89.09 ± 0.55 | <0.001 |
| BMI (kg/m^2^) | 22.08 ± 0.17 | 23.72 ± 0.16 | 24.48 ± 0.17 | 26.38 ± 0.22 | <0.001 |
| SBP (mmHg) | 110.87 ± 0.65 | 112.53 ± 0.60 | 113.58 ± 0.70 | 115.00 ± 0.69 | <0.001 |
| DBP (mmHg) | 73.43 ± 0.54 | 75.56 ± 0.55 | 75.55 ± 0.60 | 77.67 ± 0.59 | <0.001 |
| Glucose (mg/dL) | 87.47 ± 0.39 | 90.17 ± 0.44 | 90.76 ± 0.45 | 93.57 ± 0.50 | <0.001 |
| HOMA-IR | 1.33 ± 0.01 | 1.84 ± 0.01 | 2.34 ± 0.02 | 3.98 ± 0.12 | <0.001 |
| T-C (mg/dL) | 176.60 ± 1.92 | 183.30 ± 1.93 | 181.80 ± 1.84 | 192.30 ± 2.05 | <0.001 |
| TG (mg/dL) | 111.07 ± 6.05 | 136.64 ± 5.00 | 138.79 ± 6.05 | 196.14 ± 8.10 | <0.001 |
| HDL-C (mg/dL) | 53.95 ± 0.67 | 50.57 ± 0.67 | 48.90 ± 0.60 | 45.71 ± 0.56 | <0.001 |
| LDL-C (mg/dL) | 100.44 ± 1.87 | 105.41 ± 1.83 | 105.14 ± 1.78 | 107.36 ± 1.89 | 0.013 |
| Vitamin D (ng/mL) | 18.89 ± 0.38 | 18.06 ± 0.36 | 17.72 ± 0.35 | 17.84 ± 0.36 | 0.033 |
| BMD (g/cm^2^) |  |  |  |  |  |
| Total hip | 0.99 ± 0.01 | 1.00 ± 0.01 | 1.01 ± 0.01 | 1.02 ± 0.01 | 0.001 |
| Femoral neck | 0.87 ± 0.01 | 0.88 ± 0.01 | 0.89 ± 0.01 | 0.88 ± 0.01 | 0.192 |
| Femoral trochanter | 0.69 ± 0.01 | 0.69 ± 0.01 | 0.70 ± 0.00 | 0.70 ± 0.00 | 0.077 |
| Femoral intertrochanter | 1.18 ± 0.01 | 1.20 ± 0.01 | 1.22 ± 0.01 | 1.22 ± 0.01 | <0.001 |
| Lumbar spine | 0.99 ± 0.01 | 1.00 ± 0.01 | 1.01 ± 0.01 | 1.00 ± 0.01 | 0.324 |
| Whole body fat percentage | 18.43 ± 0.33 | 21.70 ± 0.34 | 22.75 ± 0.32 | 25.12 ± 0.29 | <0.001 |
| Smoking (%) | 224 (74.2%) | 229 (75.8%) | 220 (72.8%) | 223 (73.8%) | 0.867 |
| Alcohol intake (%) | 247 (81.8%) | 237 (78.5%) | 254 (84.1%) | 245 (81.1%) | 0.361 |
| Physical activity (%) | 182 (60.3%) | 175 (57.9%) | 178 (58.9%) | 168 (55.6%) | 0.700 |
| Education ≤high school (%) | 133 (44.0%) | 126 (41.7%) | 115 (38.1%) | 119 (39.4%) | 0.460 |
| House income ≤1st quartiles (%) | 25 (8.3%) | 26 (8.6%) | 25 (8.3%) | 18 (6.0%) | 0.595 |
| Females | ≤7.04 | 7.04-8.74 | 8.74-10.89 | >10.87 |  |
|  | *n*=385 | *n*=386 | *n*=386 | *n*=385 |  |
| Age (yr) | 30.75 ± 0.16 | 30.81 ± 0.16 | 30.63 ± 0.16 | 30.54 ± 0.17 | 0.255 |
| Height (cm) | 160.52 ± 0.29 | 160.56 ± 0.27 | 160.74 ± 0.30 | 160.79 ± 0.27 | 0.436 |
| Weight (kg) | 52.58 ± 0.32 | 54.41 ± 0.39 | 56.39 ± 0.40 | 62.31 ± 0.60 | <0.001 |
| WC (cm) | 70.26 ± 0.36 | 72.01 ± 0.38 | 73.65 ± 0.42 | 78.99 ± 0.55 | <0.001 |
| BMI (kg/m^2^) | 20.41 ± 0.12 | 21.09 ± 0.14 | 21.84 ± 0.15 | 24.07 ± 0.21 | <0.001 |
| SBP (mmHg) | 101.19 ± 0.48 | 102.06 ± 0.54 | 102.84 ± 0.48 | 106.62 ± 0.56 | <0.001 |
| DBP (mmHg) | 66.80 ± 0.42 | 66.63 ± 0.47 | 67.63 ± 0.43 | 70.09 ± 0.43 | <0.001 |
| Glucose (mg/dL) | 85.64 ± 0.33 | 86.98 ± 0.35 | 89.36 ± 0.34 | 92.01 ± 0.45 | <0.001 |
| HOMA-IR | 1.27 ± 0.01 | 1.70 ± 0.01 | 2.14 ± 0.01 | 3.31 ± 0.01 | <0.001 |
| T-C (mg/dL) | 170.19 ± 1.37 | 167.33 ± 1.54 | 169.39 ± 1.30 | 179.46 ± 1.57 | <0.001 |
| TG (mg/dL) | 67.35 ± 1.95 | 75.82 ± 2.26 | 88.88 ± 3.79 | 108.88 ± 3.32 | <0.001 |
| HDL-C (mg/dL) | 60.90 ± 0.61 | 58.62 ± 0.62 | 57.64 ± 0.63 | 54.00 ± 0.61 | <0.001 |
| LDL-C (mg/dL) | 95.83 ± 1.21 | 93.55 ± 1.36 | 93.97 ± 1.19 | 103.69 ± 1.45 | <0.001 |
| Vitamin D (ng/mL) | 16.97 ± 0.30 | 16.81 ± 0.31 | 16.13 ± 0.27 | 16.39 ± 0.30 | 0.069 |
| BMD (g/cm^2^) |  |  |  |  |  |
| Total hip | 0.87 ± 0.01 | 0.88 ± 0.01 | 0.89 ± 0.01 | 0.90 ± 0.01 | <0.001 |
| Femoral neck | 0.75 ± 0.01 | 0.75 ± 0.01 | 0.77 ± 0.01 | 0.77 ± 0.01 | <0.001 |
| Femoral trochanter | 0.63 ± 0.00 | 0.63 ± 0.00 | 0.64 ± 0.00 | 0.65 ± 0.00 | <0.001 |
| Femoral intertrochanter | 1.04 ± 0.01 | 1.05 ± 0.01 | 1.07 ± 0.01 | 1.08 ± 0.01 | <0.001 |
| Lumbar spine | 0.96 ± 0.01 | 0.97 ± 0.01 | 0.99 ± 0.01 | 1.00 ± 0.01 | <0.001 |
| Whole body fat percentage | 29.33 ± 0.27 | 30.90 ± 0.27 | 31.48 ± 0.28 | 34.12 ± 0.27 | <0.001 |
| Smoking (%) | 48 (12.5%) | 61 (15.8%) | 59 (15.3%) | 60 (15.6%) | 0.526 |
| Alcohol intake (%) | 204 (53.0%) | 178 (46.1%) | 196 (50.8%) | 201 (52.2%) | 0.223 |
| Physical activity (%) | 201 (52.2%) | 164 (42.5%) | 178 (46.1%) | 195 (50.6%) | 0.029 |
| Education ≤ high school (%) | 146 (37.9%) | 145 (37.6%) | 154 (39.9%) | 191 (49.6%) | 0.002 |
| House income ≤1st quartiles (%) | 18 (4.7%) | 23 (6.0%) | 22 (5.7%) | 22 (5.7%) | 0.868 |
| Labor ≥1/lifetime (%) | 48 (12.5%) | 91 (23.6%) | 89 (23.1%) | 92 (23.9%) | <0.001 |
| Use of OCs (%) | 8 (2.1%) | 6 (1.6%) | 11 (2.8%) | 4 (1.0%) | 0.290 |
| Age at menarche <12 yr (%) | 26 (6.8%) | 39 (10.1%) | 31 (8.0%) | 40 (10.4%) | 0.229 |

Data are presented as the means ± SE (standard error).

WC, waist circumference; BMI, body mass index; SBP, systolic blood pressure; DBP, diastolic blood pressure; T-C, total cholesterol; TG, triglyceride; HDL-C, high-density lipoprotein cholesterol; LDL-C, low-density lipoprotein cholesterol; BMD, bone mineral density; OCs, oral contraceptives**.**

**Supplementary Table B.**

**Unadjusted and adjusted associations between the serum fasting insulin level and bone mineral density (BMD) of the total hip, femoral neck, femoral trochanter, femoral intertrochanter, and lumbar spine in Korean males and females (*n*=2,750).**

|  | Serum fasting insulin (μU/mL) | | | | | | | | | | | | | |
| --- | --- | --- | --- | --- | --- | --- | --- | --- | --- | --- | --- | --- | --- | --- |
|  | Model 1 | | | |  | Model 2 | | | |  | Model 3 | | | |
|  | β | SE | R^2^ | *P* |  | β | SE | R^2^ | *P* |  | β | SE | R^2^ | *P* |
| Total hip (g/cm^2^) |  |  |  |  |  |  |  |  |  |  |  |  |  |  |
| All (*n*=2,750) | 0.126 | 0.000 | 0.016 | <0.001 |  | −0.065 | 0.000 | 0.389 | <0.001 |  | −0.055 | 0.001 | 0.397 | 0.001 |
| Males (*n*=1,208) | 0.063 | 0.001 | 0.004 | 0.027 |  | −0.082 | 0.001 | 0.224 | 0.004 |  | −0.068 | 0.001 | 0.237 | 0.017 |
| Females (*n*=1,542) | 0.125 | 0.001 | 0.016 | <0.001 |  | −0.054 | 0.001 | 0.227 | 0.027 |  | −0.047 | 0.001 | 0.239 | 0.061 |
| Femoral neck (g/cm^2^) |  |  |  |  |  |  |  |  |  |  |  |  |  |  |
| All (*n*=2,750) | 0.090 | 0.001 | 0.008 | <0.001 |  | −0.085 | 0.000 | 0.375 | <0.001 |  | −0.072 | 0.001 | 0.383 | <0.001 |
| Males (*n*=1,208) | 0.017 | 0.001 | 0.000 | 0.565 |  | −0.120 | 0.001 | 0.241 | <0.001 |  | −0.102 | 0.001 | 0.256 | <0.001 |
| Females (*n*=1,542) | 0.094 | 0.001 | 0.009 | <0.001 |  | −0.058 | 0.001 | 0.172 | 0.022 |  | −0.048 | 0.001 | 0.179 | 0.062 |
| Femoral trochanter (g/cm^2^) |  |  |  |  |  |  |  |  |  |  |  |  |  |  |
| All (*n*=2,750) | 0.096 | 0.000 | 0.009 | <0.001 |  | −0.064 | 0.000 | 0.244 | <0.001 |  | −0.055 | 0.000 | 0.257 | 0.003 |
| Males (*n*=1,208) | 0.024 | 0.000 | 0.001 | 0.414 |  | −0.083 | 0.000 | 0.177 | 0.004 |  | −0.070 | 0.000 | 0.189 | 0.018 |
| Females (*n*=1,542) | 0.124 | 0.000 | 0.015 | <0.001 |  | −0.035 | 0.000 | 0.196 | 0.157 |  | −0.036 | 0.000 | 0.212 | 0.156 |
| Femoral intertrochanter (g/cm^2^) |  |  |  |  |  |  |  |  |  |  |  |  |  |  |
| All (*n*=2,750) | 0.133 | 0.007 | 0.018 | <0.001 |  | −0.054 | 0.001 | 0.370 | 0.001 |  | −0.045 | 0.001 | 0.377 | 0.009 |
| Males (*n*=1,208) | 0.076 | 0.001 | 0.006 | 0.008 |  | −0.067 | 0.001 | 0.193 | 0.019 |  | −0.056 | 0.001 | 0.206 | 0.054 |
| Females (*n*=1,542) | 0.126 | 0.001 | 0.016 | <0.001 |  | −0.045 | 0.001 | 0.207 | 0.071 |  | −0.037 | 0.001 | 0.217 | 0.145 |
| Lumbar spine (g/cm^2^) |  |  |  |  |  |  |  |  |  |  |  |  |  |  |
| All (*n*=2,750) | 0.069 | 0.000 | 0.005 | <0.001 |  | −0.073 | 0.000 | 0.153 | <0.001 |  | −0.064 | 0.000 | 0.165 | 0.001 |
| Males (*n*=1,208) | 0.008 | 0.001 | 0.000 | 0.770 |  | −0.104 | 0.001 | 0.144 | <0.001 |  | −0.092 | 0.001 | 0.157 | 0.002 |
| Females (*n*=1,542) | 0.120 | 0.001 | 0.014 | <0.001 |  | −0.031 | 0.001 | 0.184 | 0.216 |  | −0.029 | 0.001 | 0.201 | 0.248 |

Model 1: Univariate linear regression analyses between the serum fasting insulin and bone mineral density (BMD) of the total hip, femur neck, femur trochanter, femur intertrochanter, and lumbar spine were conducted with no adjustments.

Model 2: Multivariate linear regression analyses between the serum fasting insulin and BMD of the total hip, femur neck, femur trochanter, femur intertrochanter, and lumbar spine were conducted after adjusting for gender, age, height, weight, and the whole body fat mass percentage.

Model 3: Multivariate linear regression analyses between the serum fasting insulin and BMD of the total hip, femur neck, femur trochanter, femur intertrochanter, and lumbar spine were conducted after adjusting for gender, age, height, weight, whole body fat mass percentage, systolic blood pressure (SBP), diastolic blood pressure (DBP), total cholesterol, triglyceride, high-density lipoprotein cholesterol (HDL-C), low-density lipoprotein cholesterol (LDL-C), vitamin D, smoking, alcohol intake, physical activity, education level, and household income in both genders as well as labor, use of oral contraceptives (OCs), and age at menarche in females.

Subgroup analyses were conducted after adjusting for previously described confounding factors according to gender using a multivariate linear regression analysis.

β, standardized regression coefficient; SE, standard error; R2, coefficient of determination.
